# A Regulated Response to Impaired Respiration Slows Behavioral Rates and Increases Lifespan in *Caenorhabditis elegans*

**DOI:** 10.1371/journal.pgen.1000450

**Published:** 2009-04-10

**Authors:** David Cristina, Michael Cary, Adam Lunceford, Catherine Clarke, Cynthia Kenyon

**Affiliations:** 1 Department of Biochemistry and Biophysics, University of California San Francisco, San Francisco, California, United States of America; 2 Department of Chemistry and Biochemistry, University of California Los Angeles, Los Angeles, California, United States of America; Huntsman Cancer Institute, United States of America

## Abstract

When mitochondrial respiration or ubiquinone production is inhibited in *Caenorhabditis elegans*, behavioral rates are slowed and lifespan is extended. Here, we show that these perturbations increase the expression of cell-protective and metabolic genes and the abundance of mitochondrial DNA. This response is similar to the response triggered by inhibiting respiration in yeast and mammalian cells, termed the “retrograde response”. As in yeast, genes switched on in *C. elegans* mitochondrial mutants extend lifespan, suggesting an underlying evolutionary conservation of mechanism. Inhibition of *fstr-1*, a potential signaling gene that is up-regulated in *clk-1* (ubiquinone-defective) mutants, and its close homolog *fstr-2* prevents the expression of many retrograde-response genes and accelerates *clk-1* behavioral and aging rates. Thus, *clk-1* mutants live in “slow motion” because of a *fstr-1/2*–dependent pathway that responds to ubiquinone. Loss of *fstr-1/2* does not suppress the phenotypes of all long-lived mitochondrial mutants. Thus, although different mitochondrial perturbations activate similar transcriptional and physiological responses, they do so in different ways.

## Author Summary

Mitochondrial respiration generates energy in the form of adenosine triphospate (ATP), a molecule that powers many cellular processes. When respiration is inhibited in *C. elegans*, rates of behavior and growth are slowed and, interestingly, lifespan is extended. In this study, we investigated the mechanism of this response. We find that inhibiting respiration increases the expression of genes predicted to protect and metabolically remodel the animal. This pattern of gene expression is reminiscent of the expression profile of long-lived respiration-defective yeast, suggesting ancient evolutionary conservation. Mutations in *clk-1*, which inhibit the synthesis of the respiratory-chain factor ubiquinone, produce gene expression, longevity, and behavioral phenotypes similar to those produced by inhibiting components of the respiratory chain. We find that knocking down the activities of two similar genes—*fsrt-1* and *fstr-2*—accelerates the behaviors and aging rates of *clk-1* mutants and inhibits the *clk-1(−)* transcriptional response. Thus, *fstr-1/2*, which encode potential signaling proteins, appear to be part of a mechanism that actively slows rates of growth, behavior, and aging in response to altered ubiquinone synthesis. Unexpectedly, *fsrt-1/2* are not required for the longevity and behavioral phenotypes produced by inhibiting the gene *isp-1*, which encodes a different component of the respiratory chain. Our findings suggest that different types of mitochondrial perturbations activate distinct pathways that converge on similar downstream processes to slow behavioral rates and extend lifespan.

## Introduction

Mitochondria generate most of the cell's energy as well as its reactive oxygen species (ROS), and mitochondrial dysfunction can cause disease and accelerate aging. Paradoxically, mitochondrial dysfunction can also increase longevity. Yeast petite mutants, which lack mitochondrial DNA and do not carry out respiration, have an increased replicative lifespan [1]. In *C. elegans*, two types of mutations that affect mitochondrial function also increase lifespan. The first type reduces respiration substantially. One such mutant, *isp-1(qm150)*, was identified in an EMS screen for mutants with delayed development and defecation rates. These animals harbor a mutation in an iron-sulfur protein in complex III of the electron transport chain and have reduced rates of oxygen consumption [2]. In addition, two independent RNA interference (RNAi) longevity screens revealed that knock-down of genes encoding components of the respiratory chain or ATP synthase decreased ATP production and rates of respiration, reduced behavioral rates and increased lifespan [3],[4]. Respiratory-chain RNAi-treated animals are smaller than *isp-1* mutants [3],[5], implying either a more severe reduction in respiration or, conceivably, a qualitatively different response. Interestingly, a mutation that reduces the level of the respiratory-chain component cytochrome c oxidase extends the lifespan of mice [6], suggesting that the underlying mechanism may be conserved in higher animals.

The second type of mitochondrial mutant is exemplified by *clk-1* mutants, which are also long lived and have reduced behavioral rates [7]. *clk-1* mutants lack a mitochondrial hydroxylase necessary for synthesis of ubiquinone, a prenylated benzoquinone required for shuttling electrons from complexes I and II to complex III during respiration [8]. Oxidative phosphorylation measurements in isolated mitochondria have shown that *clk-1* mutations reduce electron transport between complex I and III, but not between complex II and III [9]. In yeast, the *clk-1* homologue *COQ7* is necessary for respiration, and *coq7* mutants are unable to grow on non-fermentable carbon sources. In contrast, *C. elegans clk-1* mutants are not only viable, but they have nearly normal levels of respiration and ATP [10],[11]. *clk-1* mutants compensate for the lack of endogenous ubiquinone, Q_9_ (the subscript refers to the number of isoprene units) with bacterial Q_8_, provided in their diet [12],[13]. In the absence of *clk-1*, the animals accumulate the Q_9_ precursor demethoxyubiquinone (DMQ_9_). There is some debate over what role DMQ_9_ plays in the *clk-1* phenotypes, but the data suggest that they are caused by the absence of Q_9_ [13],[14],[15]. Mice with reduced levels of *Mclk-1* are also long lived [16]; though curiously, these mice do not have reduced Q_9_ levels [17].

How do these mitochondrial mutations extend lifespan? Because respiration is the major source of ROS, which could potentially accelerate aging, a simple explanation for the increased longevity of animals with reduced respiration is that they generate less ROS as the animal ages. However, timed RNAi experiments indicate that respiratory-chain activity must be reduced during development for lifespan extension. Adult-only RNAi treatments reduce ATP levels and slow behavioral rates but do not extend lifespan [3],[18]. If reducing mitochondrial respiration extended lifespan by reducing the level of ROS produced during the aging process itself, then one might expect reducing respiration at any time would extend lifespan. In addition, lifespan extension does not correlate with resistance to the oxidative stressor paraquat [4] or levels of protein carbonylation [18]. Likewise, little is known about the mechanism by which *clk-1* mutations, which have relatively small effects on respiration, extend lifespan. Overexpression of *clk-1* shortens lifespan and increases movement rates in *C. elegans* [11] suggesting that whatever the mechanism, its influence on longevity and rates of living is rate limiting in the animal.

In previous studies, mutations that suppress the slowed defecation phenotype (but not other defects) of *clk-1* mutants have been found [19]. One of these suppressor genes, *dsc-1*, has been cloned and found to a encode homeodomain protein [20]; however, in general their mode of action is not well understood. Several interesting studies have associated a *clk-1* germ-line phenotype with altered ROS signaling, and showed that *sod-1* mutations can partially suppress *clk-1* mutant's developmental delays [21],[22]. This is suggestive that *clk-1* mutations may alter ROS signaling in cells.

How might mutations affecting respiration and ubiquinone biosynthesis slow behaviors and extend the lifespan of *C. elegans*? One possibility is that these perturbations trigger a transcriptional response that alters the animal's physiology and lifespan. In yeast, loss of mitochondrial DNA is known to induce a robust transcriptional response. This change in gene expression has been called the “retrograde response”, because it implies a reversal in the normal direction of information flow between the mitochondria and nucleus [1],[23],[24]. The genes expressed during the yeast retrograde response lead to a metabolic remodeling of the cell, heat-shock resistance, and increased mitochondrial biogenesis [25],[26],[27],[28]. The retrograde response has been shown to be required for the increased longevity of these so-called yeast “petites”. Thus, lifespan extension in these yeast cells is actively regulated, and not simply a passive consequence of decreased respiration. A gene expression profile similar to the yeast retrograde response has been observed in cultured mammalian cells when mitochondrial DNA is depleted using ethidium bromide, suggesting that this transcriptional response has been conserved evolutionarily [28].

The retrograde response may be a compensatory reaction to the normal decline in mitochondrial function seen with age, since it is observed in older cells [1]. Whether it could potentially play a role in longevity determination in multicellular organisms is not known. Consistent with this possibility, *C. elegans isp-1* mutants have increased levels of expression of at least one protective gene, the superoxide dismutase *sod-3* [2]. In this study, we carried out microarray analysis of *C. elegans* mitochondrial mutants to test the hypothesis that a transcriptional response to mitochondrial perturbation slows the animal's rates of behavior and aging.

## Results

### *clk-1* Mutants Exhibit a Conserved Gene Expression Profile

*clk-1* mutants are enigmatic because they exhibit a respiration-defective behavioral and longevity phenotype without having major changes in respiration [16]. For gene expression profiling, we grew synchronized populations of *clk-1(qm30)* mutants and wild-type (N2) animals and collected them as pre-fertile adults. One thousand seven genes (listed in Supplementary Table 5) were found to be differentially expressed and were ranked using the SAM (Significance Analysis of Microarrays) tool [29] using a False Discovery Rate (FDR) of ∼0.1 as cut-off. Interestingly, the majority of the genes in this group (99%) were up-regulated relative to wild type, as was also the case in yeast petites [25],[26].

**Figure 1:**
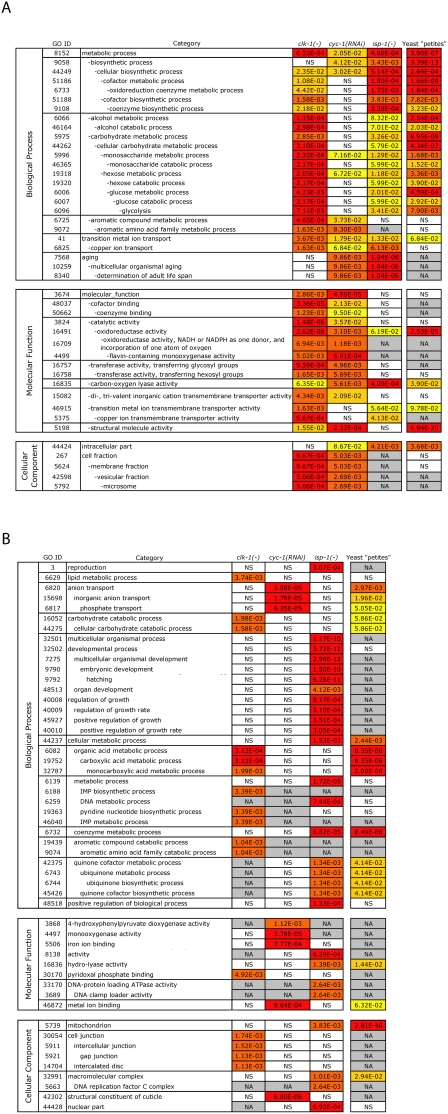
Up-regulated GO categories in worm and yeast mitochondrial mutants. Tables A and B show the p-values for the most significant GO categories found in *isp-1(−)*, *clk-1(−)* and *cyc-1(RNAi)* animals (*p*<0.1; FDR<0.1 in at least one mutant) (red represents more significant, yellow represents less significant). The “yeast petite” column indicates whether each GO category found in one or more of the *C. elegans* mutants was also overrepresented in petite yeast data sets. GO categories are aggregated into GO “branches” (same cell in table) if they are part of the same GO hierarchy and are directly related to each other. NS represents non-significant, for GO categories that were below our cut-off. NA represents not available, for GO categories that had no annotated genes in yeast or for situations in which no expression data was available for that category. GO categories were separated into two tables (A and B). A) GO categories present in two or more *C. elegans* mitochondrial mutants. All overlap sets, both between different combinations of two mutants, as well as between all three mutants, are highly significant (*p*<0.001). In addition, the number of categories shared between all three *C. elegans* mutants and yeast “petites” is also much higher than expected by random chance (*p*<0.001; one would expect 2 categories by random chance). B) GO categories present in only one mitochondrial mutant. Categories that were present in only one mutant were also less likely to be present in the yeast petite dataset. Note that cell-protective genes are not well annotated as distinct GO classes in current *C. elegans* data bases (i.e., WormBase).

The genes were assigned to Gene-Ontology (GO) categories using the software BiNGO [30], and several GO categories were found to be overrepresented (Figure 1). The nature of these categories suggested that *clk-1* mutants undergo significant metabolic reorganization and, in addition, activate a stress response similar to that elicited by xenobiotics. For example, GO categories 6006, 6007, and 6096 include genes involved in glycolysis; GO categories 16835 and 44275 include genes potentially involved in glycolysis, gluconeogenesis or anaplerotic pathways; GO category 32787 contains genes involved in anaplerotic reactions (which generate Krebs cycle intermediates); GO categories 9072, 9074 and 30170 encompass genes involved in amino acid metabolism; GO categories 6825 and 5375 include genes involved in Cu transport; GO category 6629 includes genes involved in lipid metabolism; GO category 46040 includes genes involved in nucleotide metabolism; and GO categories 4499 and 16758 include genes involved in xenobiotic response and maintenance of cellular redox state.

When we looked at individual genes, we observed up-regulation of genes encoding enzymes required for glycolysis, such as GPD-2, GPD-3, (gliceraldehyde 3-phosphate dehydrogenase), T05D4.1 (aldolase A homologue), and LDH-1 (lactate dehydrogenase). GEI-7, which is an enzyme necessary for the glyoxylate cycle in worms (see Discussion), was also up-regulated. We also observed increased expression of an isocitrate dehydrogenase, C30G12.2, likely involved in the Krebs cycle, and other alcohol dehydrogenases (*dhs-29*, *dhs-3*) that could potentially act in anaplerotic pathways. We found increased expression of proteins involved in oxidative phosphorylation, such as *asg-2* (subunit of ATP synthase complex) and F17A9.4 (NADH oxidoreductase). Also, several genes coding for enzymes involved in amino acid and nucleotide metabolism were up-regulated in *clk-1* mutants. There was also a significant increase in enzymes involved in cellular detoxification, including UDP-glycosyl transferases (UGT-53, UGT-13, UGT-43, UGT-6, UGT-39), gluthathione S-transferases (GST-4, GST-13, GST-36), superoxide dismutase (SOD-3), flavin-containing monooxygenases (FMO-1, FMO-3) and other gene classes potentially involved in xenobiotic metabolism (cytochrome P450s, alcohol dehydrogenases and ABC transporters).

To compare the transcriptional profile of *clk-1* mutants to the yeast retrograde-response genes, we referred to two previous publications studying the effects of inhibiting mitochondrial respiration in yeast petites [25],[26]. We applied BiNGO software to the most differentially expressed genes reported in those studies and compared the yeast-petite GO categories to those of *clk-1* mutants. Among the top ranking GO categories (*p*<0.1), we observed a remarkable degree of similarity (*p*<0.001) between the *clk-1* and yeast petite BiNGO categories (Figure 1).

**Figure 2:**
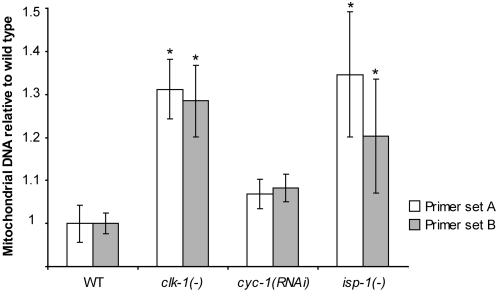
Mitochondrial DNA quantification. Percent increase in total mitochondrial DNA relative to wild type is shown, as measured by qPCR. Data are shown for two different mitochondrial primer pairs across five independent biological repeats. (*) indicates significance of *p*<0.05 after Bonferroni multiple comparison correction. Error bars are ±SEM. *clk-1(−)* primer set A: m (mean) = 1.31±0.07, primer set B: m = 1.29±0.08; *cyc-1(RNAi)* primer set A: m = 1.07±0.03, primer set B: m = 1.08±0.03; *isp-1(−)* primer set A: m = 1.35±0.14, primer set B: m = 1.21±0.13.

One of the hallmarks of the yeast response to respiration inhibition is an increase in mitochondrial biogenesis [31]. To test whether there might be an increase in mitochondrial biogenesis in *clk-1* mutants, we used Real-Time qPCR to quantify mitochondrial DNA. Total mtDNA was measured relative to genomic DNA, which provided the average number of mitochondrial genomes per cell in a population of worms (see Methods). We observed a significant increase in mitochondrial DNA levels (Figure 2). Together, these data suggest that *clk-1* mutants exhibit a response to mitochondrial dysfunction that is similar to the yeast retrograde response.

### *fstr-1/2* and *aqp-1* Contribute to the Increased Longevity of *clk-1* Mutants

**Figure 3:**
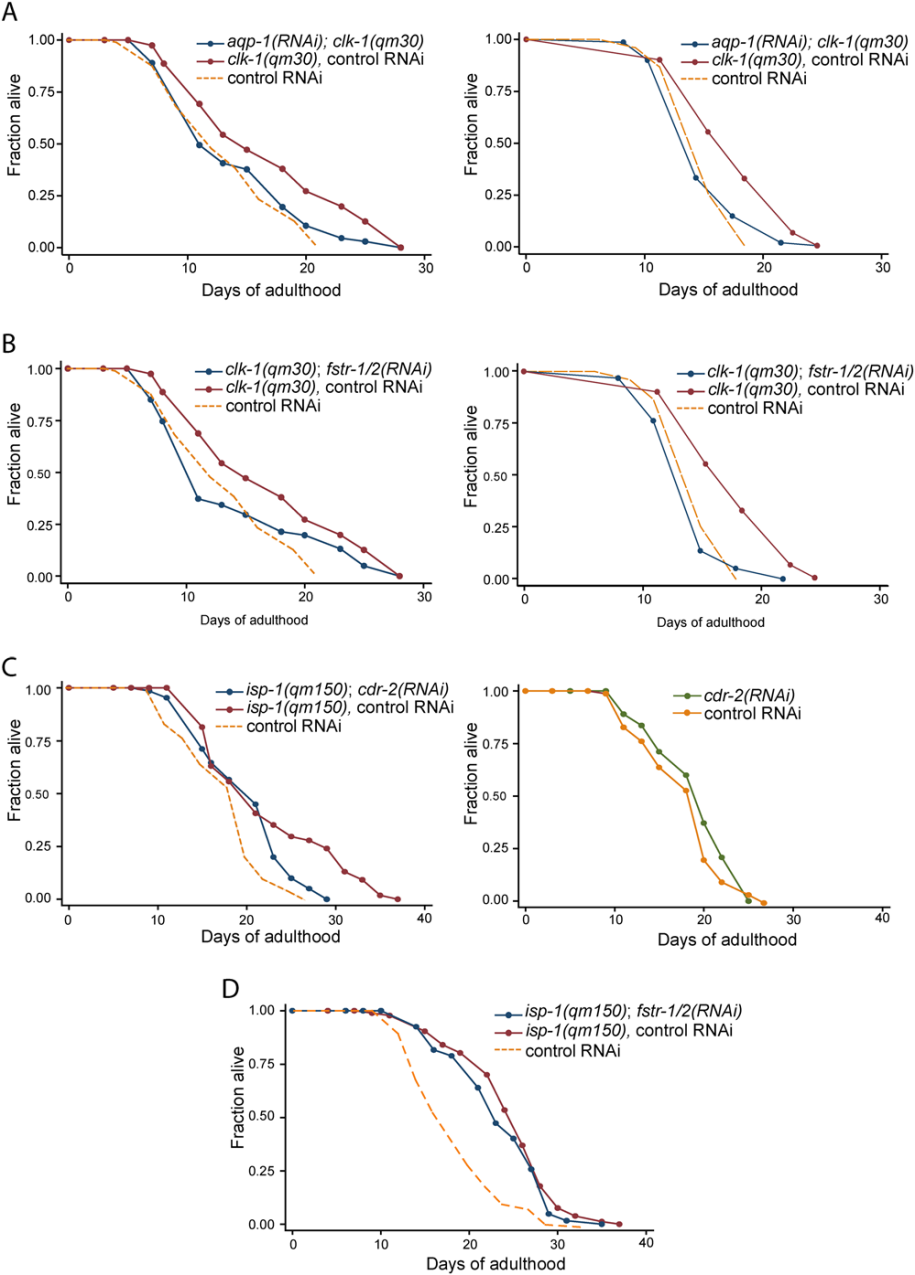
Lifespan measurements of long-lived mitochondrial mutants subjected to RNAi of individual retrograde-response genes. A) *aqp-1* RNAi significantly decreased the lifespan extension produced by *clk-1* mutations from 26% (control) to 7%; *p*<0.05 (left panel) and from 17% (control) to 0%; *p*<0.0001 (right panel). Left panel - WT subjected to control (vector-only) RNAi: N = 103, m = 13.4 days; *clk-1(−)* mutants subjected to control RNAi: N = 105, m = 16.9 days; *clk-1(−)* mutants subjected to *aqp-1* RNAi: N = 105, m = 14.4 days. Right panel - WT subjected to control (vector-only) RNAi: N = 77, m = 15.1 days; *clk-1(−)* mutants subjected to control RNAi: N = 137, m = 17.3 days; *clk-1(−)* mutants subjected to *aqp-1* RNAi: N = 70, m = 15.1 days. *aqp-1* RNAi did not significantly affect WT lifespan (Figure S1). B) *fstr-1/2* RNAi significantly decreased the lifespan extension produced by *clk-1* mutation from 26% to 3%, *p*<0.01 (left panel) and from 17% (control) to 0%; *p*<0.0001 (right panel). Left panel - WT subjected to control RNAi, same as in Figure 2A; *clk-1(−)* mutants subjected to control RNAi, same as in Figure 2A; *clk-1(−)* mutants subjected to *fstr-1/2* RNAi: N = 108, m = 13.8 days. Right Panel - WT subjected to control (vector-only) RNAi: N = 77, m = 15.1 days; *clk-1(−)* mutants subjected to control RNAi: N = 137, m = 17.3 days; *clk-1(−)* mutants subjected to *fstr-1/2* RNAi: N = 63, m = 14.5 days. *fstr-1/2* RNAi did not significantly affect WT lifespan (Figure S1). C) *cdr-2* RNAi suppressed the longevity increase produced by *isp-1* mutation from 43% to 26% (*p*<0.001) and did not significantly affect wild-type longevity. WT subjected to control RNAi: N = 90, m = 18.1 days; WT subjected to *cdr-2* RNAi: N = 87, m = 19.1 days; *isp-1(−)* mutants subjected to control RNAi: N = 89, m = 22.4 days; *isp-1 (−)* mutants subjected to *cdr-2* RNAi: N = 89, m = 20.1 days. In a second trial, *cdr-2* RNAi decreased the lifespan extension produced by *isp-1* mutation from 24% to 11% (*p*<0.001) (Table S4). Lifespans were determined at 20°C. D) *fstr-1/2* RNAi did not shorten the lifespan of *isp-1* mutants. *isp-1(−)* mutants subjected to control RNAi: N = 108, m = 24.6 days; *isp-1 (−)* mutants subjected to *fstr-1/2* RNAi: N = 104, m = 23.6 days. These experiments were repeated twice more with similar results (Table S4).

To identify genes necessary for the increased longevity of *clk-1(−)* animals, we compiled a list of differentially expressed genes from populations of L4 (late larval stage) as well as prefertile adults (see Methods). We included L4 animals in this set because previous work has shown that the L4 period is the critical period for lifespan determination in at least some mitochondrial mutants [18] (and data not shown). We picked the 75 top differentially expressed genes ranked using the SAM software package, inhibited their functions using RNAi and measured lifespan. Out of the initial list of 75, we collected lifespan data for 63 genes over two independent trials (Table S1). We established a significance cut-off of *p*<0.05 and selected RNAi clones that decreased *clk-1* longevity significantly in both trials or were statistically significant in one experiment and showed a decrease of at least 5% in the other (a 5% decrease in overall lifespan corresponds to an ∼25% decrease in the lifespan extension produced by *clk-1* mutation). We retested the positive clones in *clk-1(−)* and wild-type animals for effects on longevity (Table S2). Out of 63 RNAi clones tested, only two decreased *clk-1* mutant longevity in all three trials (Figure 3A and 3B). Neither of these clones significantly shortened wild-type lifespan, suggesting that they may play a role specifically in *clk-1* mutant lifespan (Figure S1). One of these clones corresponded to *aqp-1*, which encodes a glycerol channel [32]. *aqp-1* RNAi decreased the lifespan extension that would normally be produced by *clk-1* mutations from 26% to 7% (*p*<0.05) and from 17% to 0% (*p*<0.0001), and did not affect wild-type longevity in two separate experiments (Figure S1). Interestingly, *aqp-1* (also called *dod-4*) has already been shown to contribute to the long lifespan of *daf-2* insulin/IGF-1-receptor mutants [33]. The other RNAi clone, corresponding to a gene we call *fstr-1* (for “faster”, also known as *gfi-1*) decreased the lifespan extension produced by *clk-1* mutations from 26% to 3% (*p*<0.01) and from 17% to 0% (p<0.0001) while not affecting wild-type lifespan in two separate experiments. The effects of *aqp-1* RNAi and *fstr-1* RNAi on the longevity of *clk-1* mutants were tested three times, with consistent results, although the extent of suppression varied between experiments (Tables S1 and S2, Figure S1). We examined the genome for the possibility that *fstr-1* RNAi might cross-inhibit another gene, and found that the RNAi clone was likely to knock down a close homolog (with 96% protein sequence identity) located next to *fstr-1* that did not exhibit *clk-1*-dependent regulation in our microarray analysis. We call this gene *fstr-2*, and henceforth we refer to their combined functions, as inferred from RNAi, as *fstr-1/2* function.

### *fstr-1/2* Knockdown Increases the Behavioral Rates of *clk-1* Mutants

**Figure 4:**
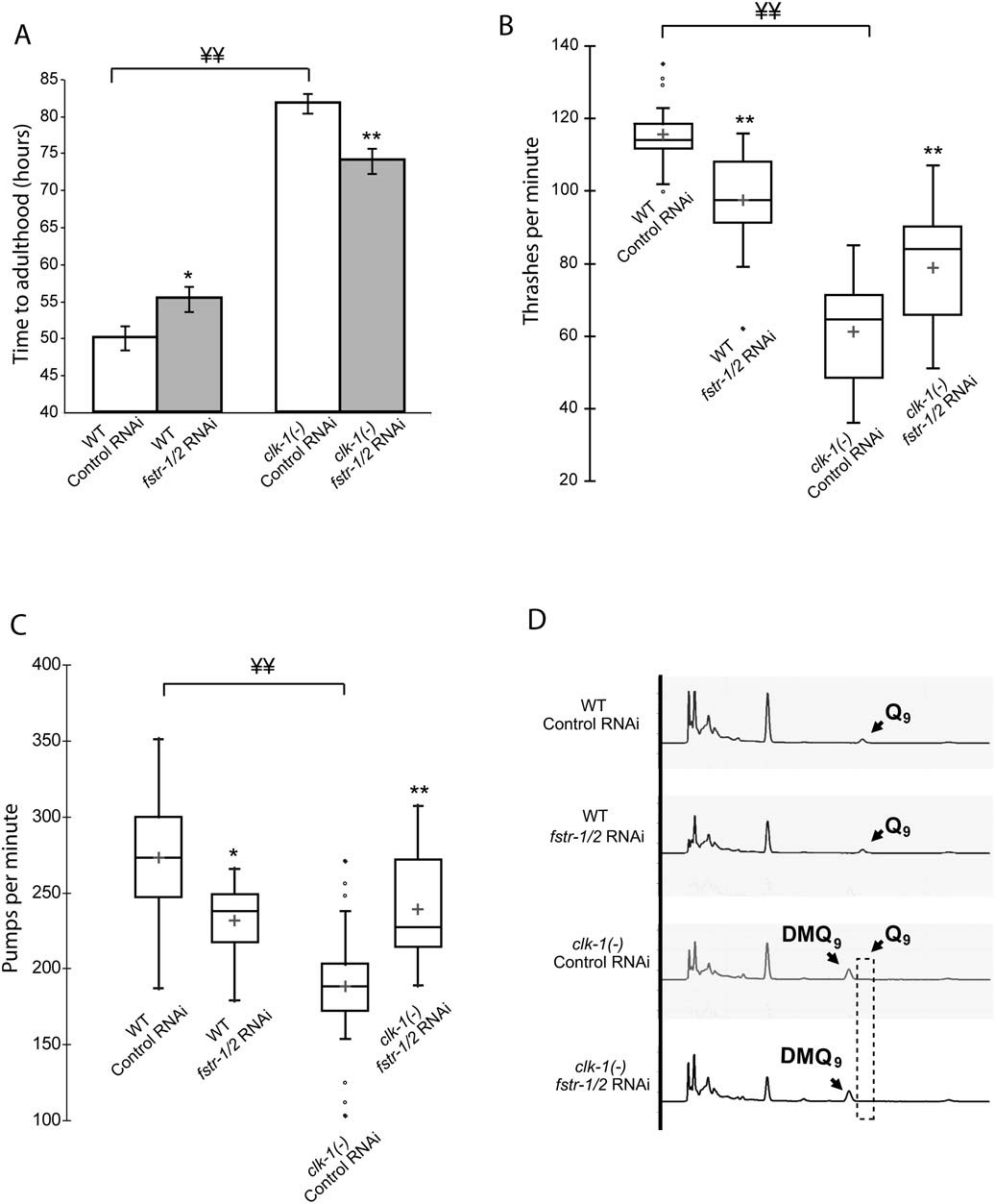
*fstr-1/2* RNAi speeds up *clk-1(−)* animals. A) Time to adulthood. *clk-1* mutants take much longer to reach adulthood than wild-type animals. This rate of development is accelerated by *fstr-1/2* RNAi. In contrast, *fstr-1/2* RNAi slowed the growth rate of wild type. These effects were very robust and were observed in every experiment. WT subjected to control RNAi: 47.4±1.6 hours; WT subjected to *fstr-1/2* RNAi: 53.2±1.7 hours; *clk-1(−)* mutants subjected to control RNAi: 82.2±1.2 hours; *clk-1(−)* mutants subjected to *fstr-1/2* RNAi: 72±1.7 hours. B) Boxplots illustrating thrashing rates measured on day 3 of adulthood. *fstr-1/2* RNAi treatment significantly reduced the average thrashing rate of wild-type animals and significantly increased the average thrashing rate of *clk-1(−)* mutants. WT subjected to control RNAi: 115.6±3.1 thrashes/min; WT subjected to *fstr-1/2* RNAi: 97.5±4.3 thrashes/min; *clk-1(−)* mutants subjected to control RNAi: 61.2±5 thrashes/min; *clk-1(−)* mutants subjected to *fstr-1/2* RNAi: 78.9±5.7 thrashes/min. C) Boxplots illustrating pumping rates. *fstr-1/2* RNAi decreased the average pumping rate of wild type and increased the pumping rate of *clk-1(−)* mutants. WT subjected to control RNAi: 273.3±41.1 pumps/min; WT subjected to *fstr-1/2* RNAi: 231.8±24.7 pumps/min; *clk-1(−)* mutants subjected to control RNAi: 188.5±41 pumps/min; *clk-1(−)* mutants subjected to *fstr-1/2* RNAi: 238.9±36.4 pumps/min. In Figs. 3A, 3B and 3C, error bars depict SEM; * depicts a significance of *p*<0.05 when compared to controls, ** depicts a significance of *p*<0.008 when compared to controls, ¥¥ depicts a significance of *p*<0.008. *P*<0.008 is the cut-off set by the Bonferroni correction for multiple comparisons. D) HPLC analysis of quinone content. The chromatograms show a representative run of three independent experiments for each of the different conditions (WT subjected to control RNAi, WT subjected to *fstr-1/2* RNAi, *clk-1(−)* mutants subjected to control RNAi and *clk-1(−)* mutants subjected to *fstr-1/2* RNAi). The Q_9_ peak is absent from *clk-1*(−) animals and instead the intermediate DMQ_9_ peak is present. *fstr-1/2* RNAi had no effect on Q_9_ levels in wild-type or *clk-1(−)* mutants, in *clk-1* mutants Q_9_ levels remained below the detection threshold.

In addition to increased longevity, the most striking phenotypes of *C. elegans* mitochondrial mutants are their decreased behavioral rates. In principle, these rates could decrease as a direct consequence of impaired mitochondrial function. Alternatively, it is possible that their slowed behavioral rates reflect a regulated response to mitochondrial perturbation; simply speaking, they slow down to conserve energy. To look for genes that slow the behaviors of *clk-1* mutants, we inhibited the top 100 up-regulated genes from microarrays of L4 and pre-fertile adults using RNAi and measured time it took for L1 larvae to develop to adulthood. We found that knockdown of *fstr-1/2* in *clk-1* mutants consistently increased the rate of growth to adulthood (Figure 4A). This phenotype was most striking when the animals were examined 75–80 hours after hatching. At this time, no control *clk-1(−)* mutants had reached adulthood, whereas 95–100% of the *fstr-1/2* RNAi treated animals were adults. *fstr-1/2* RNAi treatment also increased the behavioral rates of *clk-1(−)* animals, as measured by thrashing and pumping (Fig 4B and 4C). These effects were not observed in wild type; in fact, in wild type, knock-down of these genes had the opposite effect, slowing development and decreasing rates of thrashing and pumping.

To test whether *fstr-1/2* RNAi somehow restored wild-type *clk-1* function, we examined ubiquinone profiles. Using HPLC, we observed the expected decrease in UQ_9_ and increase in DMQ_9_ in *clk-1* mutants, and we observed the same mutant pattern of ubiquinone species in *clk-1* mutants subjected to *fstr-1/2* RNAi (Figure 4D). Thus, *fstr-1/2* RNAi suppresses the phenotypes of animals that still have an altered, Clk-1(−), pattern of ubiquinone species. This suggests that the wild-type *fstr-1/2* gene slows behavior and extends lifespan in response to the changes in ubiquinone produced by *clk-1* mutations.

### *fstr-1/2* Is Necessary for Gene Expression Changes in *clk-1* Mutants

**Figure 5:**
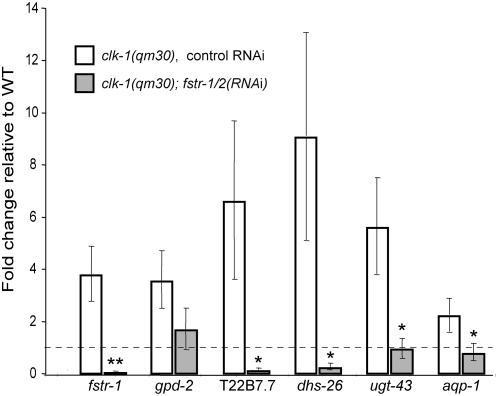
*fstr-1/2* RNAi inhibits expression of *clk-1* retrograde-response genes. The graph shows the effect of *fstr-1/2* RNAi on the degree of up-regulation of the five individual *clk-1* retrograde-response genes we examined, as determined by quantitative RT-PCR. (**) represents a significance of *p*<0.01, (*) represents a significance of *p*<0.05, in comparing mRNA levels from *clk-1(−)*; *fstr-1/2(RNAi)* animals vs. *clk-1(−)* control animals. Each bar represents the average of 3 independent biological repeats. Error bars are ±SEM. *clk-1(−)* animals subjected to control RNAi showed significantly higher expression than did wild type subjected to control RNAi (*p*<0.05) for all genes tested. With the exception of *gpd-2*, all genes showed a significant decrease in expression (*p*<0.05) in the presence of *fstr-1/2* RNAi. Note that *gpd-2* shared the trend, with p = 0.061, and was clearly affected when assayed using the p*gpd-2::gfp* reporter *in vivo* (Figure 7 and Figure S5).

Next we asked whether FSTR-1/2 modulates the *clk-1* mutant phenotype by influencing the retrograde response. Using real-time qPCR, we looked at the effects of *fstr-1/2* RNAi on the expression levels of five of the genes whose expression was most significantly up-regulated in the microarrays: *gpd-2*, a glyceraldehyde 3-phosphate dehydrogenase involved in glycolysis; T22B7.7, an Acyl-CoA thioesterase, involved in anaplerotic reactions; *dhs-26*, an alcohol dehydrogenase; *ugt-43*, an UDP-glucoronosyl transferase; and the aquaporin gene *aqp-1*. The qPCR data confirmed the microarray studies, in that all of these genes were up-regulated in *clk-1* mutants. We found that *fstr-1/2* RNAi significantly and consistently decreased expression of these genes in a *clk-1*(−) background (Figure 5) but not in wild type (Figure S2). Thus, the gene expression changes observed in *clk-1* mutants are at least partially dependent on *fstr-1/2*. Together these findings suggest that in *clk-1* mutants, *fstr-1/2(+)* decreases rates of behavior and extends lifespan by triggering downstream changes in gene expression.

### *fstr-1(+)* May Act in the Intestine and/or Nervous System to Slow Down *clk-1* Mutants

**Figure 6:**
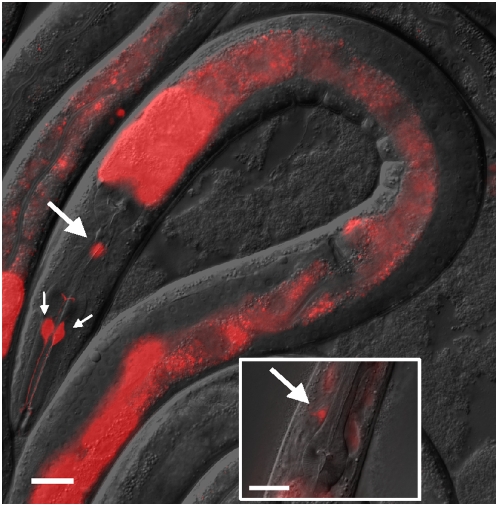
*fstr-1* is expressed in the intestine and three neurons. The figure shows the expression of a p*fstr-1::mCherry* promoter fusion in *clk-1(−)* mutants. The transgene is expressed in the intestine, especially in anterior cells, as well as in RIH and I1L/R neurons. Small arrows point to cell bodies of I1L/R neurons, with their characteristic processes visible; large arrows point to the cell body of the RIH neuron. The small panel shows a more detailed view of RIH neuron. Scale bar represents 20 µm. We observed a similar, but fainter, mCherry distribution in a wild-type background (not shown).

Because *fstr-1* is up-regulated in *clk-1* mutants, we were particularly interested to learn where in the animal it was expressed. To investigate this, we generated transgenic animals expressing the fluorescent protein mCherry under the control of the *fstr-1* promoter. We observed strong expression in three neurons located in the head and throughout the intestine, particularly in the anterior intestinal cells (Figure 6). We identified the three neurons as RIH and I1L/R. RIH is a nerve-ring interneuron of unknown function and I1L/R are pharyngeal interneurons that regulate pharyngeal pumping rates in response to touch and removal of bacteria. We saw the same pattern of expression in the *clk-1*(−) mutant and wild-type, but the intensity of expression was increased in the mutant, consistent with our qRT-PCR and microarray data. Together these findings suggest that *fstr-1* acts in the intestine and/or in specific neurons to slow the rates of aging and behavior in *clk-1* mutants.

### A Similar Gene-Expression Profile in Animals with Reduced Respiration

To compare the pattern of gene expression in *clk-1* mutants to that of respiration mutants that have more strongly reduced levels of oxygen consumption and ATP, we performed microarray analysis of *isp-1(qm150)* and *cyc-1(RNAi)* animals. (*cyc-1* encodes cytochrome c reductase, which is a component of complex III of the electron transport chain.) We grew synchronized populations of *isp-1(qm150)* mutants and *cyc-1* RNAi-treated animals in parallel with wild-type control animals, collected them as young, pre-fertile adults and analyzed the resulting microarray data as described above for *clk-1* mutants. The SAM algorithm with a false discovery rate of ∼0.1 yielded 814 significant genes for *isp-1(−)* mutants and 7662 significant genes for *cyc-1(RNAi)* animals. (For the complete list, please see Tables S6 and S7.) Thus, it seems that *cyc-1* RNAi induces a broader transcriptional response than do *clk-1* and *isp-1* mutations, which correlates with the increased severity of the Cyc-1(RNAi) phenotype. In addition, *cyc-1* RNAi-treated animals, when compared to *isp-1* and *clk-1* mutants, showed increased expression of additional cell-protective genes, including chaperones (*hsp-6*, *hsp-70*), superoxide dismutases (*sod-4*, *sod-3*) and xenobiotic detoxification enzymes (*ugt-2*, *ugt-47*, *ugt-36*, *gst-8*, *gst-22*, *gst-24*, *dhs-5*, *dhs-28*). Interestingly, other genes encoding detoxification enzymes were down regulated, possibly implying the deployment of a specific detoxification program. Using BiNGO analysis, we identified several GO categories that were overrepresented in each mutant (Figure 1). A highly significant fraction of the top GO categories (*p*<0.1) was shared between either two, or all three, of the mutant strains (*p*<0.001; Figure 1). By chance, one would expect 2 of the 10 GO categories shared by all three mitochondrial mutants and annotated in yeast to also be significant in the yeast petite cells. In contrast, we observed 8 categories in common (*p*<0.001).

In addition to identifying GO categories, we looked for individual genes that were expressed in a similar way in the three *C. elegans* mitochondrial mutants. We found a highly significant (*p* = 7.19E-15) overlap set of 73 differentially-expressed genes (Table S3). In addition, we observed an increase in mitochondrial DNA levels in *isp-1* mutants and a smaller increase in *cyc-1(RNAi)* animals that did not reach statistical significance (*p* = 0.354) (Figure 2). Taken together, these data suggest that the gene expression profiles of different *C. elegans* mitochondrial mutants are similar to one another and to the yeast retrograde response.

### *cdr-2* RNAi Suppresses the Increased Longevity of *isp-1* Mutants

Since *isp-1(−)*, *clk-1(−)* and *cyc-1(RNAi)* animals are all long-lived, gene expression patterns that are shared between all three might be particularly likely to contribute to lifespan extension. Double RNAi experiments in *C. elegans* can be difficult to interpret, so we did not attempt RNAi knockdowns in *cyc-1(RNAi)* animals. However, we did attempt to knock down the top thirty statistically-significant shared genes, individually, in an *isp-1* background. (Of these, 21 were not present in the *clk-1* set we described above, which contained only the top 75 differentially expressed genes). We obtained data for all of these genes (Table S4). Of these, only one RNAi clone, *cdr-2*, consistently made our significance cut-off [*p*<0.05 and a 10% decrease in *isp-1* mutant longevity, which corresponds to a 50% decrease in the lifespan extension produced by the *isp-1* mutation.] *cdr-2* RNAi reduced the lifespan extension produced by the *isp-1* mutation from 43% (control RNAi) to 26% (*p*<0.001), while not affecting wild-type longevity (Figure 3C).

*cdr-2* encodes a member of the glutathione S-transferase family. These enzymes catalyze the conjugation of reduced glutathione to electrophilic centers on different substrates. This activity contributes to detoxification of both endogenous toxins and xenobiotics, suggesting that the increased longevity of *isp-1(−)* mutants is at least partially dependent on a cellular detoxification response. We note that in one of our three trials, *cdr-2* RNAi significantly shortened the lifespan extension produced by *clk-1* mutation (from 21% to 14%). This finding suggests that *cdr-2* may be involved more generally for lifespan extension in mitochondrial mutants.

### *fstr-1/2*'s Regulatory Function Is Specific to *clk-1* Mutants

Given the remarkable reversal of the *clk-1* mutant phenotype by *fstr-1/2* RNAi, we were interested in examining its function in the respiratory-chain mutants. We found that *fstr-1* was significantly up-regulated in *isp-1* mutants but, unexpectedly, not in *cyc-1(RNAi)* animals. Using RNAi, we asked whether *fstr-1/2* might influence the behavioral phenotypes of *isp-1(qm150)* mutants. We restricted our analysis to lifespan and time to adulthood because *isp-1* mutants did not move often enough to provide consistent behavioral rates. We found that the developmental rates of *isp-1* mutants were severely decreased in the presence of *fstr-1/2* RNAi, leading to developmental arrest of many animals. We found that *fstr-1/2* RNAi had no effect on the lifespan of *isp-1* mutants that reached adulthood (Figure 3D). Thus the effect of *fstr-1/2* RNAi on *isp-1* mutants was more similar to the effect of *fstr-1/2* RNAi on wild type than to its effect on *clk-1* mutants.

**Figure 7:**
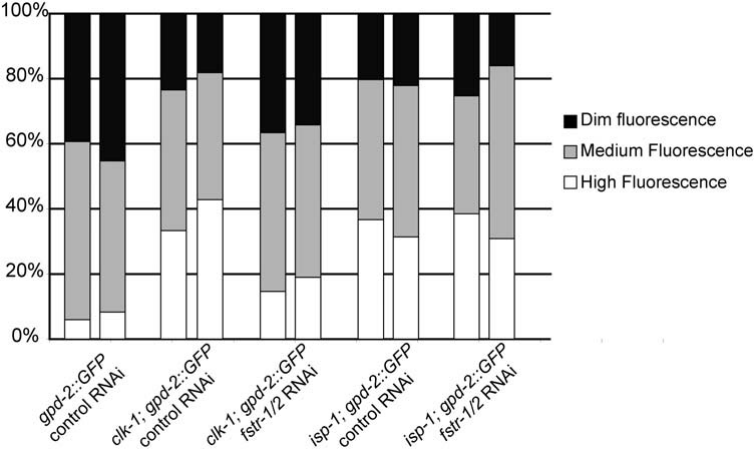
*fstr-1/2* RNAi does not affect *gpd-2* expression in an *isp-1* mutant. Worms harboring a p*gpd-2::gfp* promoter fusion were ranked as: Dim Fluorescence, Medium Fluorescence and High Fluorescence based on visual inspection. Each bar represents a different population (N = 80 worms). Two separate observations were made for each condition. See Figure S5 for representative high- and dim-fluorescence populations.

We wanted to know whether *fstr-1/2* was necessary for gene expression changes in an *isp-1(−)* background, but because *isp-1* mutants subjected to *fstr-1/2* RNAi grew very slowly and asynchronously, we could not use qRT-PCR. Instead, we assayed gene expression *in vivo* by introducing the *isp-1* mutation into a strain expressing GFP under the control of the *gpd-2* promoter, which drives expression of a glycolysis gene that is up-regulated by these mitochondrial mutations (Figure 7). We found that *fstr-1/2* RNAi prevented the up-regulation of this reporter in a *clk-1* background but not in an *isp-1* background. Together, these data suggest that the Isp-1(−) and Clk-1(−) behavioral and longevity phenotypes are established by distinct mechanisms.

## Discussion

*C. elegans* respiration mutants appear to live in “slow motion”, as the rates of a wide variety of processes, including rates of growth to adulthood, aging and behavior, are all reduced. This phenotype is shared by *clk-1* ubiquinone-biosynthetic mutants, which exhibit only a mild and temporary decrease in overall rates of respiration, and have normal ATP levels [10]. This spectrum of behavioral and longevity phenotypes is not seen in the many *C. elegans* mutants whose longevity requires the transcription factor DAF-16/FOXO, or in calorically restricted animals, so these mitochondrial mutants appear to comprise a distinct class of longevity mutants. Reducing *clk-1* activity extends the lifespan of mice, as does reducing cytochrome c oxidase levels, suggesting that a better understanding of these mutants could potentially have implications for human health and longevity.

In this study, we used microarray analysis and RNAi to ask whether a transcriptional response to mitochondrial perturbation might cause these distinctive behavioral and longevity phenotypes.

### A *C. elegans* Retrograde Response

**Figure 8:**
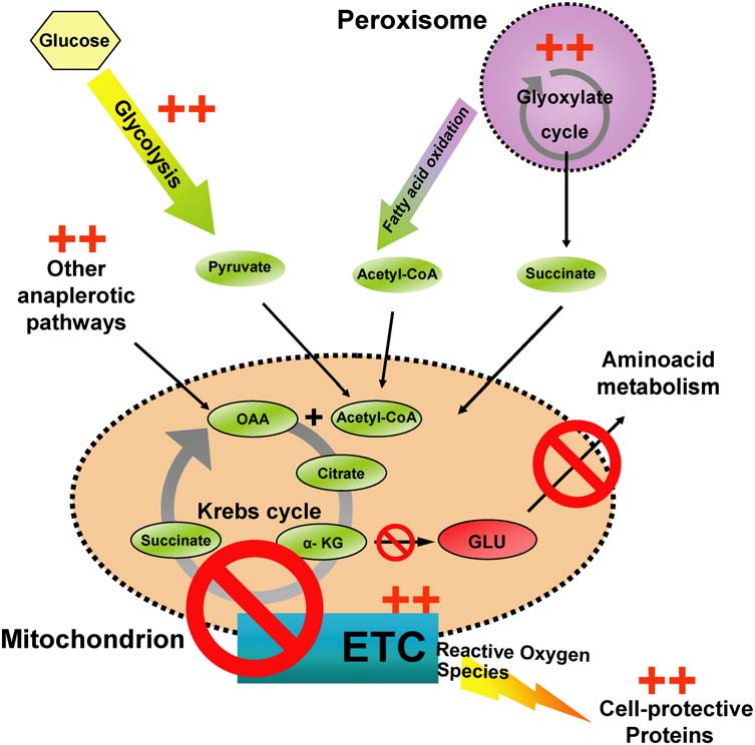
*C. elegans* retrograde response. The picture diagrams metabolic changes thought to occur during the yeast retrograde response (Butow and Avadhani 2004). “++” indicates that these same changes are observed in the *C. elegans* retrograde response. Impairment of electron flow during oxidative phosphorylation is predicted to have two effects. The first is an increase in ROS, due to increased likelihood of electrons transferring to free oxygen (Lenaz 2001). Consistent with increased ROS stress (which we did not assay directly), we observed an increase in cell-protective genes. Secondly, the Krebs cycle is disrupted because the enzyme succinate dehydrogenase is an integral part of both the electron transport chain and of the Krebs cycle. When succinate dehydrogenase oxidizes succinate into fumarate, it feeds electrons into the ETC, and when this flow is blocked, the enzyme's activity is inhibited. The Krebs cycle is necessary for synthesis of glutamate, which in turn is required for amino acid metabolism. Impairment of the electron transport chain leads to decreased glutamate production. Possibly in order to counteract this decrease in glutamate production, the cell induces anaplerotic pathways that feed intermediates into the Krebs cycle, thus allowing production of glutamate. Under conditions of impaired respiration, glycolytic gene expression increases, consistent with glycolysis' becoming a major source of ATP. Consistent with a compensatory response, expression of genes involved in oxidative phosphorylation is up-regulated. OAA, Oxaloacetate; GLU, glutamate; ETC, electron transport chain; α-KG, α-ketoglutarate.

Gene expression profiling of these mutants was quite revealing, because each of their expression profiles exhibited striking similarity to the yeast retrograde response. The yeast retrograde response, which also lengthens lifespan, appears to remodel the cell's metabolism. Without respiration, the Krebs cycle cannot be completed, as succinate cannot be oxidized to fumarate (Figure 8). This prevents the formation of oxaloacetate (OAA), which in turn decreases the availability alpha-ketoglutarate, which is the precursor of glutamate, an essential metabolite in amino acid metabolism. In order to generate precursors of glutamate, respiration-deficient cells must activate alternative (anaplerotic) pathways that supply the mitochondria with OAA and acetyl-CoA [27]. Activation of anaplerotic pathways was observed in respiration-defective yeast [25],[26] and human cells [28].

Our microarrays reveal transcriptional activation of genes encoding several metabolic enzymes that have roles in anaplerotic reactions, such as the glyoxylate cycle and fatty acid oxidation. The glyoxylate cycle occurs mainly in the peroxisomes and bypasses the succinate-to-fumarate step of the Krebs cycle through the formation of glyoxylate, eventually leading to the formation of succinate, which can be fed back into the Krebs cycle (Figure 8). We observed increased expression of the gene encoding the major *C. elegans* glyoxylate-cycle enzyme, GEI-7, in the three mitochondrial mutants we examined. We did not observe an RNAi phenotype for this clone in the *clk-1* mutant; however, because a *gei-7* mutant was available, we examined *cyc-1(RNAi)*; *gei-7* animals and found a large suppression of the *cyc-1(RNAi)* longevity phenotype, decreasing lifespan extension from 80% to 15% with little effect on wild type (Figure S4). Malate dehydrogrenases catalyze synthesis of OAA from malate, which is also an important step in recycling Krebs cycle intermediates. F46E10.10 encodes a malate dehydrogenase and is significantly up-regulated in all three long-lived mitochondrial mutants we examined. Fatty-acid oxidation provides acetyl-CoA, which feeds into the Krebs cycle by reacting with OAA to form citrate. This pathway is activated in long-lived yeast lacking mitochondrial DNA [27]. We also detected increased expression of several genes that are involved in fatty acid oxidation. *clk-1(−)* animals exhibited increased expression of *acs-2* (acetyl-CoA synthetase ) and *fat-6* (fatty acid desaturase); and *isp-1* mutants exhibited increased expression of T02G5.4 (acetyl-CoA thiolase) and T05G5.6 (enoyl-CoA hydratase). The expression profiles of *cyc-1(RNAi)* animals, however, contained fewer significant genes involved in fatty acid oxidation, suggesting there may be some differences in metabolic adjustments between different mitochondrial mutants. Interestingly, recent work has shown that long-lived mice with decreased levels of *Mclk-1* have an increase in α-ketoglutarate dehydrogenase activity, consistent with an up-regulation of the Krebs cycle [17]. Furthermore, the animals have a decrease in overall NAD levels, which in itself could hamper normal Krebs cycle activity and further decrease glutamate synthesis [26].

In all three long-lived mitochondrial mutants, we observed a significant increase in expression of genes involved in glycolysis. This was expected, since glycolysis becomes a more important source of ATP when oxidative phosphorylation is inhibited.

In addition to these metabolic shifts, we also observed increased expression of a significant number of stress response genes in all three mitochondrial mutants, ranging from genes increasing xenobiotic drug resistance to protein chaperones. This is consistent with previous *in vitro* observations that impairment of electron flow during oxidative phosphorylation is actually likely to generate more ROS [34], and suggests these animals may be responding to this additional cellular insult.

Finally, in all three strains, genes involved in the oxidative phosphorylation process itself were up-regulated, and we observed increased levels of mitochondrial DNA in *clk-1* and *isp-1* mutants. Thus, apparently *C. elegans* mitochondrial mutants, like yeast petites [26], attempt to compensate for reduced levels of respiration. Together these findings indicate that the transcriptional response triggered by conditions that inhibit respiration in *C. elegans* is similar to that triggered in yeast, and suggest the presence of a conserved underlying mechanism for lifespan extension.

While this manuscript was in preparation, Falk *et al.* reported the gene expression pattern of a mixture of long-lived and short-lived respiration-defective mutants compared to wild type [35]. In the future, it will be interesting to learn whether the expression patterns of short-lived mitochondrial mutants differ from those of the long-lived mutants. The lifespan of one such short-lived mutant, *mev-1(kn1)* was increased when respiration was lowered further using respiratory-chain RNAi (Figure S3), arguing that their short lifespans are not due simply to insufficient respiratory-chain activity.

### Long-Term Reductions in Respiration Are Not Necessary to Maintain Expression of the Retrograde Response

It was interesting to find that *clk-1* mutations trigger a conserved transcriptional response even though they only have a mild effect on respiration. There are at least two possible interpretations for this finding. The first is that the transcriptional response to *clk-1* mutation need not be triggered by reduced respiration itself, but instead can be triggered by signals that are generally associated with reduced respiration, such as fluctuations in ubiquinone levels. Such fluctuations could have acquired the ability to induce the retrograde response during evolution because they allowed the animal to conserve energy in the face of a perceived energy shortage. Alternatively, perhaps the *clk-1* mutation does inhibit respiration more severely initially, but the physiological changes elicited by the retrograde response restore the steady-state level of respiration closer to normal.

### The Retrograde Response Is Probably Required for the Longevity of *C. elegans* Mitochondrial Mutants

In yeast, the retrograde response is induced via helix-loop-helix transcription factors that do not appear to be present in *C. elegans*. When the genes encoding these transcription factors are deleted in yeast, inhibiting respiration does not induce the retrograde response, and lifespan is not extended [1]. Thus, in yeast, the retrograde response likely increases lifespan. Our data suggest that this is the case in this multicellular animal as well. First, inhibiting the activity of at least some of the genes up-regulated in mitochondrial mutants was sufficient to shorten their lifespan without obviously affecting the lifespan of wild type. In particular, the glutathione S-transferase gene *cdr-2* was up-regulated in all of the long lived mutants and it contributed to lifespan extension consistently in *isp-1* respiration-defective mutants and, at least in some trials, in *clk-1* mutants as well. This finding suggests that the prominent cell-protective gene expression response that we observe contributes to longevity. In addition, the metabolic shifts we observed are also likely to influence lifespan, as the longevity of *cyc-1(RNAi)* animals required the glyoxylate-cycle gene *gei-7*, and the longevity of *clk-1* mutants was promoted by the glycerol channel *aqp-1*.

Our failure to observe effects on lifespan with most of the RNAi clones we tested does not necessarily mean that they do not influence lifespan (though this may be the case). It seems possible that many of these genes could act cumulatively to influence lifespan. The lifespan extension of *clk-1* and *isp-1* mutants was only ∼20% in most experiments, so only perturbations that are fairly strong would be visible in our assays. In general, although we have no reason to discount the importance of genes whose knockdowns produced statistically significant effects on lifespan, because the magnitude of the effects were small, we remain cautious in our interpretation.

The second argument for the importance of the *C. elegans* transcriptional response in the longevity of mitochondrial mutants comes from our studies of *fstr-1/2*. *fstr-1* was up-regulated in *clk-1* mutants, and this gene, and/or its constitutively-expressed homolog *fstr-2*, is required, in turn, for a robust transcriptional retrograde response. Knocking down *fstr-1/2* activity with RNAi did not suppress the primary ubiquinone defect. However, none of the five up-regulated genes we tested was up-regulated in the presence of *fstr-1/2* RNAi. These genes included metabolic as well as cell-protective genes, arguing that *fstr-1/2* may be a major regulator of the retrograde response in *clk-1* mutants. This restoration of a normal transcriptional profile correlated with a suppression of the behavioral, growth and longevity phenotypes of *clk-1* mutants. Together all of these findings support the hypothesis that a conserved mitochondrial retrograde response extends lifespan in metazoans as well as in yeast. We note, however, that *fstr-1/2* RNAi had stronger effects on the induction of the retrograde-response genes we tested than it had on the *clk-1* behavioral phenotypes. This suggests either that part of the retrograde response is expressed independently of *fstr-1/2*, or that mechanisms that do not involve transcription also influence the *clk-1* phenotype.

### The Function of FSTR-1/2

How does FSTR-1/2 regulate gene expression? Little is known about the molecular function of FSTR-1/2. The predicted FSTR-1 and FSTR-2 proteins contain 21 ET modules, which are domains of unknown function containing 8–10 conserved cysteines predicted to form 4–5 disulphide bridges and a C-terminal putative transmembrane domain. Sequence alignment studies using the BLAST algorithm showed weak similarities to a secreted yeast protein (*AGA1*) and a predicted mouse membrane protein (Zonadhesin). We also looked for structural homologues of *fstr-1/2* using the software package PHYRE [36] and found highly significant predicted structural similarities to portions of ErbB1/2/3/4. ErbB proteins belong to a highly conserved family of receptor tyrosine kinases that play many roles in cell biology and disease. Members of the ErbB family usually contain an extracellular region (∼620 amino acids) that recognizes and binds ligands, a single membrane spanning region and an intracellular tyrosine kinase domain. However, we did not find a predicted tyrosine kinase domain or a secretion signal in *fstr-1/2*'s sequence, which makes a potential connection to the ErbB family unclear. Finally, FSTR-1 (originally called GFI-1, for GEX-interacting protein) was identified in a two-hybrid screen as a potential binding partner of UNC-68, a muscle-specific *C. elegans* ryanodine receptor. Our attempts to find phenotypic similarities or functional interactions between these two genes were unsuccessful (data not shown), so their potential relationship remains unclear.

Our finding that FSTR-1 is expressed in the intestine (which is *C. elegans* entire endoderm, including its site of fat storage), as well as a small number of neurons, raises the possibility that these two tissues are particularly important for the response to mitochondrial perturbation. Interestingly, the glycerol channel *aqp-1* is expressed exclusively in the intestine and pharynx [32], further implicating the intestine in the *clk-1* longevity pathway. In the future, it will be very interesting to learn how FSTR-1 and FSTR-2 function at the molecular level to initiate a response to altered ubiquinone levels.

### Different Paths to a Similar Phenotype

**Figure 9:**
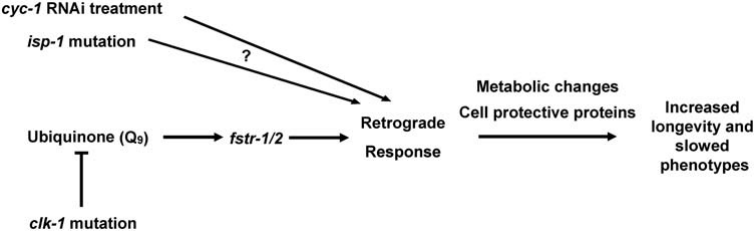
*C. elegans* mitochondrial mutants activate similar transcriptional responses in different ways. In this model, *clk-1* mutation decreases ubiquinone levels, which increases *fstr-1* expression (possibly with the assistance of *fstr-2*, which is expressed constitutively). *fstr-1/2* are then necessary for expression of other genes that are part of the retrograde response, which in turn are likely to be responsible for the longevity and slowed phenotypes. *clk-1*, *isp-1* and *cyc-1(RNAi)* mutants show similar transcriptional responses, however, *fstr-1/2* is necessary for the transcriptional response of *clk-1* but not *isp-1* mutants.

Long-lived *C. elegans* mitochondrial mutants share many phenotypes; however, there are also some significant differences between them. As mentioned above, they differ in their respiration rates, ATP levels and body size. In addition, respiratory-chain RNAi and *clk-1* mutations both extend the lifespans of *daf-2* insulin/IGF-1 receptor mutants [3],[7], whereas *isp-1* mutations do not [2] (DC and CK, unpublished). These differences have prompted the question of how similar the *C. elegans* mitochondrial mutants really are to each other [5]. Our microarray observations suggest that the overall nature of the response is similar, though the specific genes affected and the extent to which they are activated varies between mitochondrial mutants. Perhaps these differences are phenotypically significant; for example, *clk-1* mutants may have near normal respiration rates because they can compensate more fully than the other mutants to a primary respiratory-chain defect. On the other hand, there is a clear difference in the regulation of the *clk-1* and *isp-1* mutant phenotypes, since *fstr-1/2* is necessary for the *clk-1* mutant phenotypes but not for the *isp-1* mutant phenotypes (Figure 9). It is possible that there exist yet-unidentified regulators that control the transcriptional response to mitochondrial perturbation in all of these mutants, thus unifying their phenotypes. In any case, it will be interesting to learn how the *isp-1* and *cyc-1* retrograde responses are regulated and at what point these pathways converge to control the same downstream genes.

## Methods

### Strains

The strains used in this study were: N2-Bristol (WT), *fer-15(b26)*; *fem-1(hc17)*, *clk-1(qm30)* [37], *isp-1(qm150)* [2], *gei-7(ok531)*, *mev-1(kn1)* [38], *muEx491*[*pfstr-1*::*mCherry*+p*odr-1*::*cfp*], *clk-1(qm30)*; *muEx491*, *sEx11128*[p*gpd-2*::*gfp*], *isp-1(qm150)*; *sEx11128*[p*gpd-2*::*gfp*]. All strains used except for *mev-1(kn1)* were outcrossed to our laboratory's wild-type N2 strain 4 times.

### Microarray Hybridizations

We constructed microarrays using single-strand DNA oligos representing 20,374 unique *C. elegans* genes. These were purchased from Illumina [39]. Populations were starvation-synchronized as L1s overnight and collected at two different times: as L4s staged based on vulval morphology and as pre-fertile adults soon after the L4-to-adult molt, to guarantee maximum synchronicity between animals that grew to adulthood at different rates. Hybridizations were performed using standard techniques described in [33]. Total RNA was purified using TriZol reagent, mRNA was purified using Oligotex (Qiagen) and cDNA was labeled using Cy-dyes prior to hybridization. The chips described are direct comparisons between N2 and either *clk-1(qm30)* or *isp-1(qm150)* animals; or between *fer-15(b26)*; *fem-1(hc17)* animals subjected to control (vector only) RNAi versus *cyc-1* RNAi. The animals were harvested as L4 larvae or young (pre-fertile) adults. We performed four independent biological repeats for each condition with the exception of *clk-1(−)* and *isp-1(−)* L4-staged populations, where we only collected data for two biological repeats. Dye-swaps and technical repeats were averaged and analyzed as one biological repeat. Scanning was done using a GenePix 4000B scanner, and initial spot quality check was done using Genepix 6.0 software. During the analysis we used two different sets of chips for each mutant: the “combined set” includes a combination of all L4 and adult chips, and the “adult-only set” only includes chips from populations collected as adults.

### Significance Analysis

The microarray data were analyzed twice over the several-year period spanned by these studies. The initial analysis, used to generate candidate genes that may be functional in the extended longevity of mitochondrial mutants, was performed on the combined set (L4 and adults) of microarrays. In this analysis, standard ratio-based normalization and default program settings for flagging missing or “bad” spots were used in the Acuity 4.0 software package. Gene significance was calculated using the SAM software package. All of the lifespan analysis described herein was based on genes at the top of the list when the data were analyzed in this way.

The second analysis, used to generate genes for use in the comparative GO analysis, was performed on the adult-only set of arrays. These data were renormalized using lowess as well as ratio-based normalizations. On the assumption that genes within the same operon should in general have similar expression patterns, flagging parameters were adjusted to those that maximized the expression correlation of genes within the same operon on a “training” subset of the arrays. Genes not present in at least three of the arrays were not considered. These data were analyzed using the SAM software package and genes were considered significant below a FDR (false discovery rate) of 0.1.

### Gene Set Overlap Analysis

An algorithm, which we named the “*p-q* algorithm”, was designed and implemented in the Python programming language to determine the set of genes that are differentially regulated in all three combined (L4 and adult) microarray data sets. The algorithm takes as input a set of microarray hybridization data and estimates the *q-value* for each gene using the method described in [40] and *p-values* estimated using Student's t-test. It then iterates through each *q-value* and calculates the probability of seeing the observed number of genes that would be significant in all three data sets, should that *q-value* be used as the threshold for significance. The algorithm reports the set of genes that overlap between the three data sets at the *q-value* cut-off that achieved maximum overlap significance, as well as the probability of seeing such a degree of overlap by random chance. Probabilities are calculated using the hypergeometric distribution when possible, or the Poisson approximation when necessary.

### GO Analysis

GO categories were found using the BINGO software starting from a list of differentially expressed genes obtained from running SAM on the set of adult-only microarrays, with a significance cut-off of FDR< = 0.1 for each *C. elegans* mutant. Yeast GO categories were obtained by analyzing a dataset that we constructed pooling the differentially-expressed genes from two different publications [25],[26].

### RNAi

Bacterial feeding RNAi experiments were performed as described previously [33]. Clones were picked from Julie Ahringer RNAi library and were all verified by DNA sequencing.

### Survival Measurements

Lifespan analysis was conducted as previously described [3]. All assays were done at 25°C unless otherwise stated. The lifespan measurements depicted in Supplementary Table 1 were done in the presence of 20 mM FUDR (fluorodeoxyuridine) to inhibit progeny growth. The Stata 8.0 software package (Stata Corporation) was used for statistical analysis and to calculate means and percentiles. In all cases p-values were calculated using the logrank (Mantel-Cox) method.

### Mitochondrial DNA Quantification

Mitochondrial DNA was quantified using Real Time-qPCR. We used two primer sets for mitochondria DNA graciously provided by Dana Miller from the Roth lab at the Fred Hutchinson Cancer Research Center:

Mito1 Forward: GTTTATGCTGCTGTAGCGTG, Reverse: CTGTTAAAGCAAGTGGACGAG; Mito2- Forward: CTAGGTTATATTGCCACGGTG, Reverse: CAATAAACATCTCT-GCATCACC. The results were normalized to genomic DNA using a primer pairs specific for *ama-1* and *nhr-23*: *ama-1*- Forward: TGGAACTCTGGAGTCACACC, Reverse: CATCCTCCTT-CATTGAACGG; *nhr-23* – Forward: CAGAAACACTGAAGAACGCG, Reverse: CGATCTGCAGTGAATAGCTC. Animals were grown and collected as described above for microarray studies and lysed in a standard buffer containing proteinase K for 1 hour at 65°C. qPCR was performed using SYBR GREEN PCR Master Mix (Applied Biosystems). Each comparison pools 5 biological repeats. Results were normalized to wild type using 7300 System SDS Software.

### Rates of Growth and Behavior

Time to adulthood was measured as time (±2 hours) at which 95% of animals reached adulthood. Measurements shown represent pooled data from five independent experiments, error bars represent SEM. Pumping rate was measured as the average number of pharyngeal pumps per minute (n = 10) over three independent trials. Thrashing rate was measured as the average number of body thrashes in M9 buffer in one minute (n = 10) over three independent trials. All measurements were conducted on day three of adulthood.

### Quantitative RT-PCR

Real-time RT-PCR was carried out using the 7300 Real Time PCR System (Applied Biosystems, Foster City, CA, USA). Primers and probes were designed specifically for each gene using Primer3 software.

### Construction of the P*fstr-1*::mCherry Promoter Fusion

To generate p*fstr-1*::*mCherry*-expressing animals, a p*fstr-1::mCherry* construct was made using the Invitrogen Gateway Cloning technology. The promoter was amplified from genomic DNA using a primer set obtained from Mark Vidal's online promoterome database (Forward: ggggacaactttgtatagaaaagttgaggccagctttagataat; Reverse: ggggactgcttttttgtacaaacttgtcatctgaaatttgaatgtgttagt). The construct obtained was sequenced and injected as described (Mello and Fire, 1995) at 10 ng/µl into N2 animals to generate a transgenic line (indicated by *muEx491* designation). The coinjection marker P*odr-1::gfp* was injected at 50 ng/µl.

## Supporting Information

Figure S1 Lifespan measurements of long-lived mitochondrial mutants subjected to RNAi of individual retrograde-response genes. A. *aqp-1* RNAi did not significantly affect WT longevity. WT subjected to control (vector-only) RNAi: N = 103, m = 13.4 days; WT subjected to *aqp-1* RNAi: N = 106, m = 13.4 days. B. *aqp-1* RNAi significantly decreased the lifespan extension produced by *clk-1* mutations from 33% (control) to 20%; p<0.001. WT subjected to control (vector-only) RNAi: N = 81, m = 14.0 days; *clk-1(−)* mutants subjected to control RNAi: N = 85, m = 18.6 days; *clk-1(−)* mutants subjected to *aqp-1* RNAi: N = 81, m = 16.8 days. C. *fstr-1/2* RNAi did not significantly affect WT longevity. WT subjected to control (vector-only) RNAi: N = 103, m = 13.4 days; WT subjected to *fstr-1/2* RNAi: N = 105, m = 13.9 days. D. *fstr-1/2* RNAi significantly decreased the lifespan extension produced by *clk-1* mutations from 33% (control) to 26%; p<0.05. WT subjected to control (vector-only) RNAi: N = 81, m = 14.0 days; *clk-1(−)* mutants subjected to control RNAi: N = 85, m = 18.6 days; *clk-1(−)* mutants subjected to *fstr-1/2* RNAi: N = 78, m = 17.8 days.
(0.63 MB TIF)

Figure S2 Retrograde-response genes respond differently to *fstr-1/2* RNAi in wild-type animals and *clk-1 (−)* mutants. For gene expression patterns in a *clk-1* background, see Figure 5.
(0.18 MB TIF)

Figure S3 The short lifespan of *mev-1* mutants is increased by respiratory-chain RNAi. WT subjected to *cyc-1* RNAi: N = 81, m = 27.6 days; WT subjected to control RNAi: N = 81, m = 19.7 days; *mev-1(kn1)* mutants subjected to *cyc-1* RNAi: N = 80, m = 16.7 days; *mev-1(kn1)* mutants subjected to control RNAi: N = 78, m = 13.2 days. This lifespan analysis was performed twice, p<0.001 both times.
(1.22 MB TIF)

Figure S4 The glyoxylate cycle gene *gei-7* is partially necessary for *cyc-1* RNAi to increase longevity. WT subjected to *cyc-1* RNAi: N = 109, m = 40.1 days; WT subjected to control RNAi: N = 113, m = 22.3 days; *gei-7(ok531)* mutant subjected to *cyc-1* RNAi: N = 118, m = 26.6 days; *gei-7(ok531)* mutant subjected to control RNAi: N = 119, m = 22.1 days. This lifespan analysis was performed twice, p<0.001 both times.
(1.14 MB TIF)

Figure S5 *fstr-1/2* RNAi treatment decreases *gpd-2::gfp* expression in a *clk-1* mutant. Since there is significant variability within populations, all our scoring for Figure 5 was done by observing populations, not individual worms. Panel A represents a population scored as high fluorescence in Figure 5 and panel B represents a population scored as dim fluorescence. Populations with intermediate brightness were scored medium fluorescence. A. Image depicts *clk-1; gpd-2::gfp* animals subjected to control RNAi. B. Image depicts *clk-1; gpd-2::gfp* animals subjected to *fstr-1/2* RNAi.
(1.95 MB TIF)

Table S1 RNAi inhibition of the top 75 up-regulated genes in *clk-1* mutants. Out of 75, we successfully completed two lifespans for 63 genes, over 4 different time periods (each experiment is represented by a different color). A. Table S1A shows the control lifespans for each experiment plotted as percent *clk-1(−)* longevity difference relative to WT (2nd line) or percent WT longevity difference relative to *clk-1(−)* (4th line). B. Table S1B includes the lifespan data for the different RNAi experiments and is shown as percent longevity difference relative to *clk-1(−)*. Calculated as: *clk-1(−)* control longevity minus RNAi treatment longevity divided by *clk-1(−)* control longevity.
(0.03 MB XLS)

Table S2 RNAi clones that consistently shortened the lifespan of *ckl-1* mutants but not wild type. Out of 7 RNAi clones tested a third time, two, *fstr-1/2* RNAi and *aqp-1* RNAi, decreased *clk-1* mutant longevity while having no effect on WT in two separate trials.
(0.02 MB XLS)

Table S3 The most significant differentially-expressed genes overlapping between *isp-1*, *clk-1* and *cyc-1(RNAi)* mutants. The set of overlapping genes was determined using the “*p-q*” algorithm (see Methods), using the combined set of L4 and adult microarray data as input. Significance values shown were calculated using the SAM software package, also run on the combined set of L4 and adult data. The top 30 genes listed were tested for suppression of lifespan extension in *isp-1* mutants (see Table S4). Also shown are the SAM significance values for these genes using the adult-only set of microarrays.
(1.34 MB XLS)

Table S4 Genes tested for suppression of the extended lifespan of *isp-1* mutants. We tested 31 genes for suppression of the increased longevity of *isp-1* mutant increased longevity using a cut-off of p<0.05 and 10% overall decreased longevity. The RNAi clones that made the cut-off were retested. (Each experiment is represented by a different color). A. Table S4A shows the control lifespans for each experiment plotted as percent *isp-1(−)* longevity difference relative to WT (2nd line) or percent WT longevity difference relative to *isp-1(−)* (4th line). B. Table S4B includes the lifespan data for the different RNAi experiments and is shown as percent longevity difference relative to *isp-1(−)*. Calculated as: *isp-1(−)* control longevity minus RNAi treatment longevity divided by *isp-1(−)* control longevity. C. Table S4C represents retests done in a wild-type background.
(0.02 MB XLS)

Table S5 Significant genes from *clk-1(−)* microarrays using SAM algorithm with an FDR≈0.1 from adult-only chips. SAM plot attached, plotting observed distribution versus randomized distribution.
(0.86 MB XLS)

Table S6 Significant genes from *isp-1(−)* microarrays using SAM algorithm with an FDR≈0.1 from adult-only chips. SAM plot attached, plotting observed distribution versus randomized distribution.
(1.04 MB XLS)

Table S7 Significant genes from *cyc-1(RNAi)* microarrays using SAM algorithm with an FDR≈0.1 from adult only chips. SAM plot attached, plotting observed distribution versus randomized distribution.
(2.47 MB XLS)
