# Supplementary figures and images for: A Regulated Response to Impaired Respiration Slows Behavioral Rates and Increases Lifespan in Caenorhabditis elegans
Source: PLoS Genet. 2009 Apr 10;5(4):e1000450. doi: 10.1371/journal.pgen.1000450 (PMC2660839; doi:10.1371/journal.pgen.1000450)

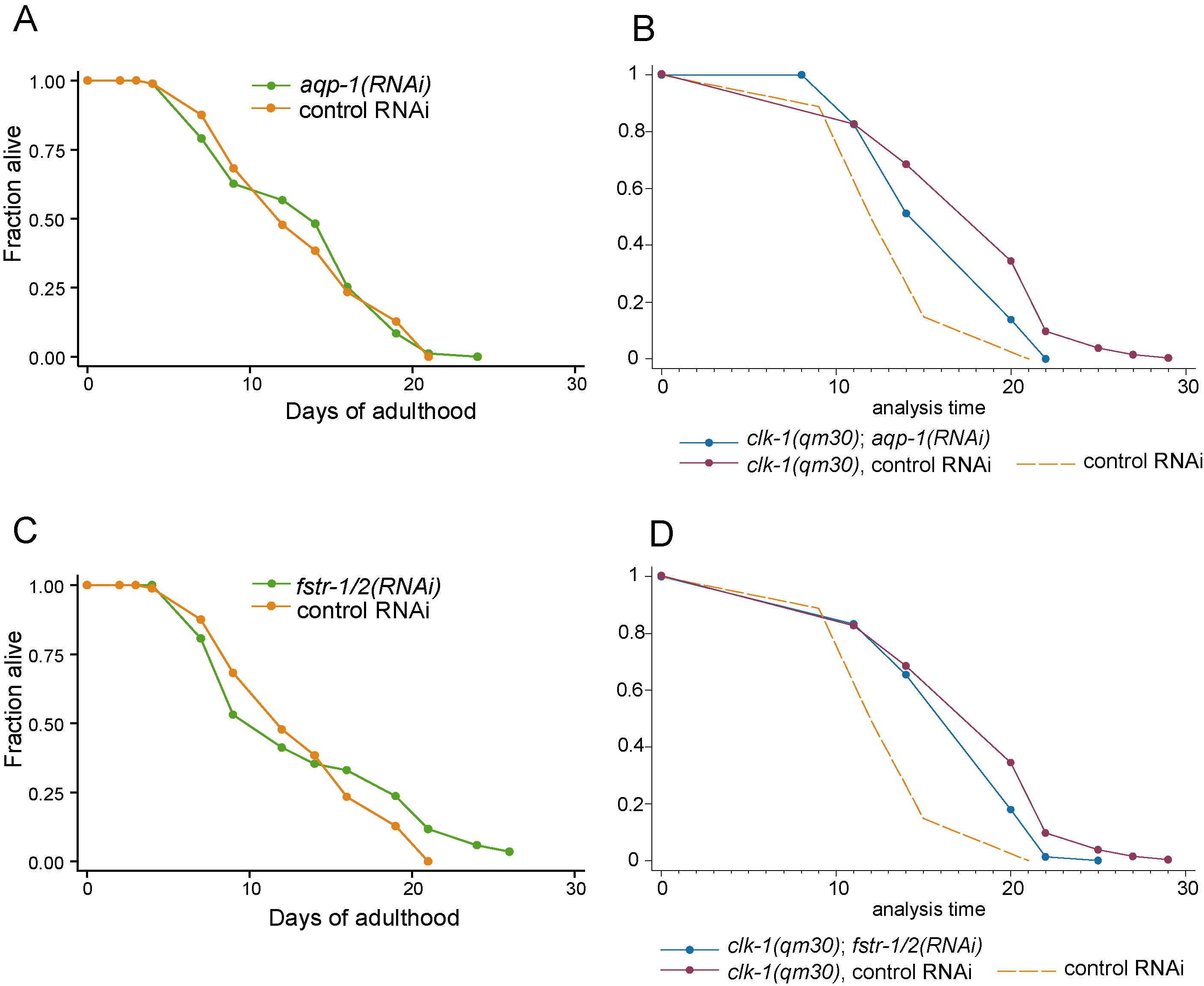

Supplement: Figure S1 — Lifespan measurements of long-lived mitochondrial mutants subjected to RNAi of individual retrograde-response genes. A. aqp-1 RNAi did not significantly affect WT longevity. WT subjected to control (vector-only) RNAi: N = 103, m = 13.4 days; WT subjected to aqp-1 RNAi: N = 106, m = 13.4 days. B. aqp-1 RNAi significantly decreased the lifespan extension produced by clk-1 mutations from 33% (control) to 20%; p<0.001. WT subjected to control (vector-only) RNAi: N = 81, m = 14.0 days; clk-1(−) mutants subjected to control RNAi: N = 85, m = 18.6 days; clk-1(−) mutants subjected to aqp-1 RNAi: N = 81, m = 16.8 days. C. fstr-1/2 RNAi did not significantly affect WT longevity. WT subjected to control (vector-only) RNAi: N = 103, m = 13.4 days; WT subjected to fstr-1/2 RNAi: N = 105, m = 13.9 days. D. fstr-1/2 RNAi significantly decreased the lifespan extension produced by clk-1 mutations from 33% (control) to 26%; p<0.05. WT subjected to control (vector-only) RNAi: N = 81, m = 14.0 days; clk-1(−) mutants subjected to control RNAi: N = 85, m = 18.6 days; clk-1(−) mutants subjected to fstr-1/2 RNAi: N = 78, m = 17.8 days. (0.63 MB TIF) [file pgen.1000450.s001.tif]

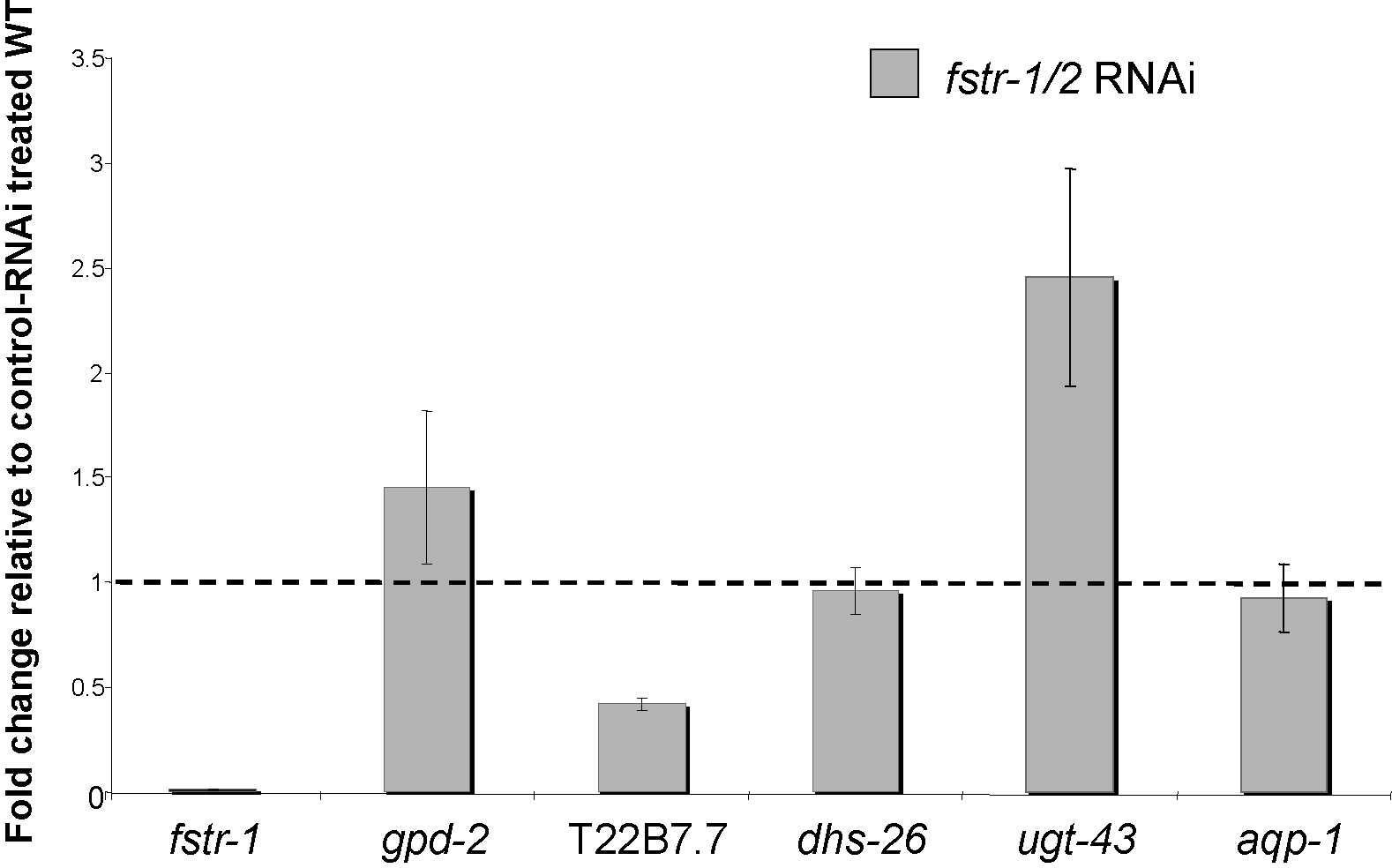

Supplement: Figure S2 — Retrograde-response genes respond differently to fstr-1/2 RNAi in wild-type animals and clk-1 (−) mutants. For gene expression patterns in a clk-1 background, see Figure 5. (0.18 MB TIF) [file pgen.1000450.s002.tif]

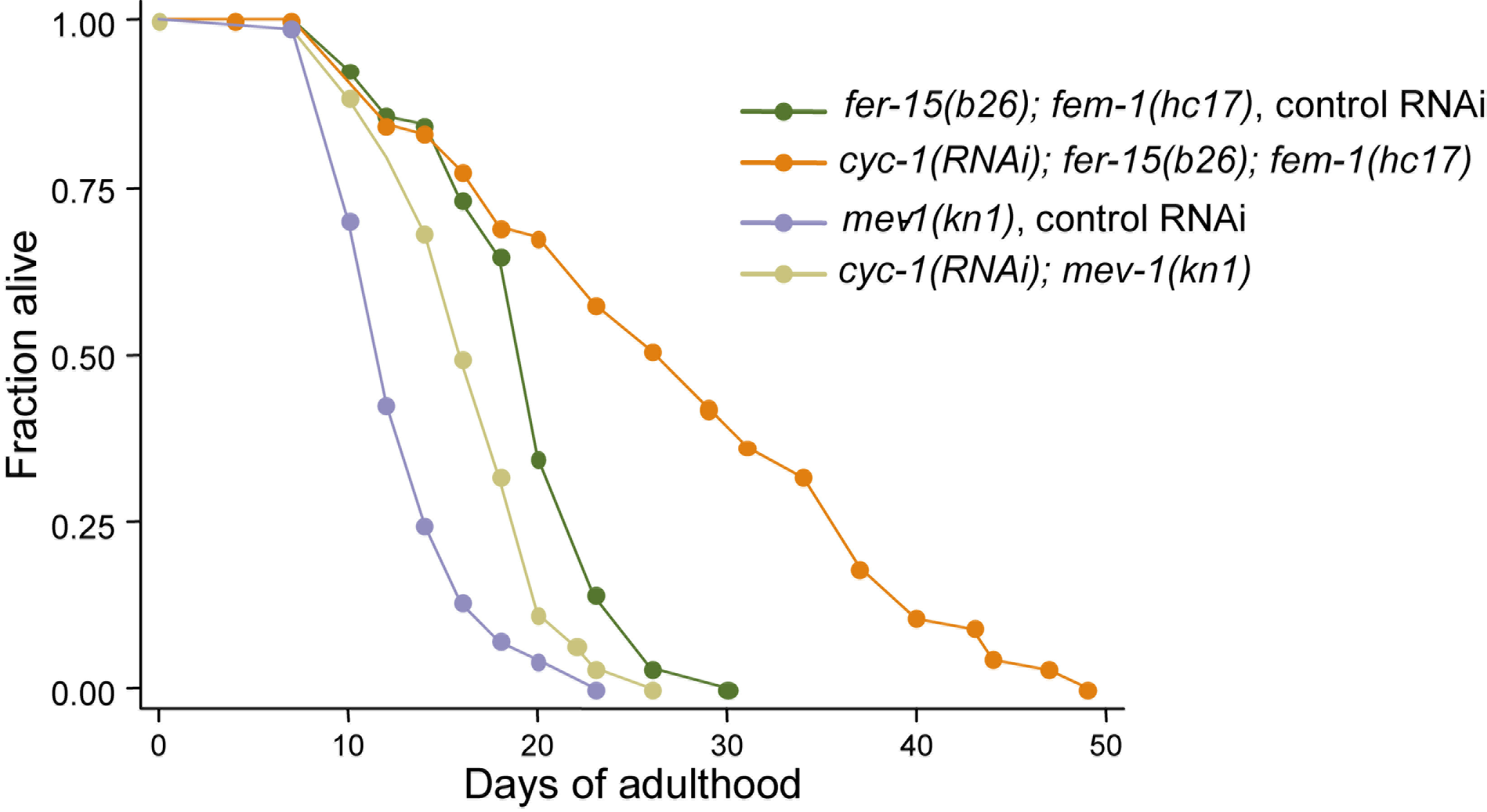

Supplement: Figure S3 — The short lifespan of mev-1 mutants is increased by respiratory-chain RNAi. WT subjected to cyc-1 RNAi: N = 81, m = 27.6 days; WT subjected to control RNAi: N = 81, m = 19.7 days; mev-1(kn1) mutants subjected to cyc-1 RNAi: N = 80, m = 16.7 days; mev-1(kn1) mutants subjected to control RNAi: N = 78, m = 13.2 days. This lifespan analysis was performed twice, p<0.001 both times. (1.22 MB TIF) [file pgen.1000450.s003.tif]

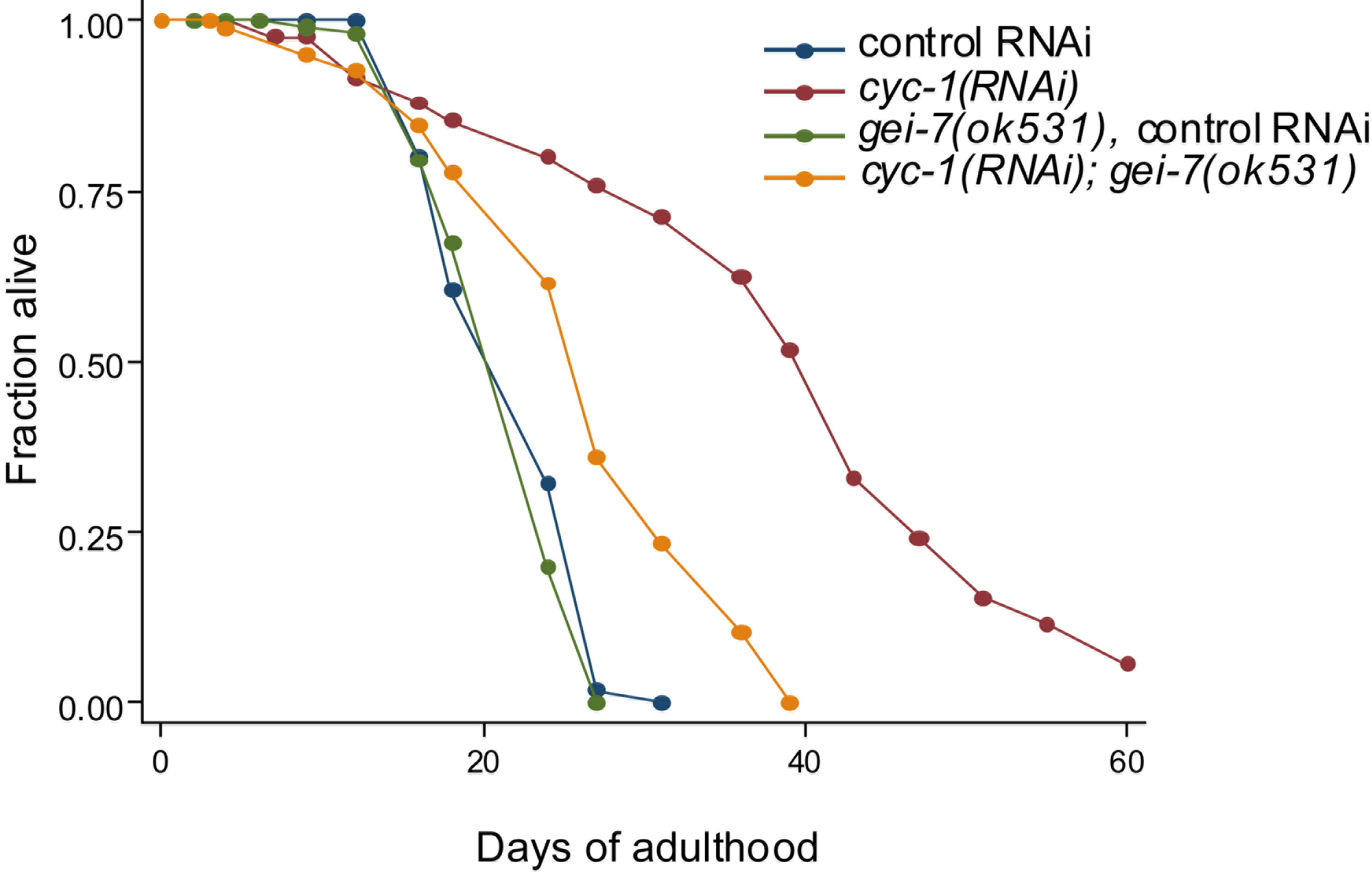

Supplement: Figure S4 — The glyoxylate cycle gene gei-7 is partially necessary for cyc-1 RNAi to increase longevity. WT subjected to cyc-1 RNAi: N = 109, m = 40.1 days; WT subjected to control RNAi: N = 113, m = 22.3 days; gei-7(ok531) mutant subjected to cyc-1 RNAi: N = 118, m = 26.6 days; gei-7(ok531) mutant subjected to control RNAi: N = 119, m = 22.1 days. This lifespan analysis was performed twice, p<0.001 both times. (1.14 MB TIF) [file pgen.1000450.s004.tif]

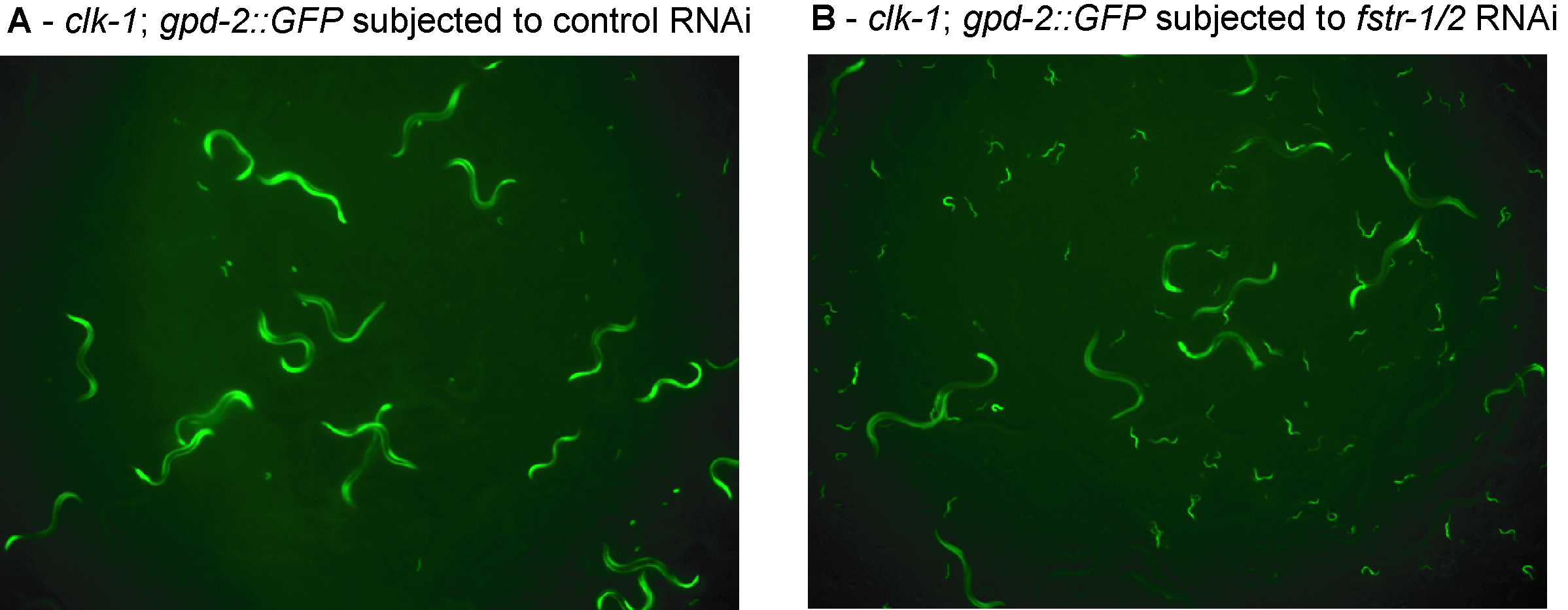

Supplement: Figure S5 — fstr-1/2 RNAi treatment decreases gpd-2::gfp expression in a clk-1 mutant. Since there is significant variability within populations, all our scoring for Figure 5 was done by observing populations, not individual worms. Panel A represents a population scored as high fluorescence in Figure 5 and panel B represents a population scored as dim fluorescence. Populations with intermediate brightness were scored medium fluorescence. A. Image depicts clk-1; gpd-2::gfp animals subjected to control RNAi. B. Image depicts clk-1; gpd-2::gfp animals subjected to fstr-1/2 RNAi. (1.95 MB TIF) [file pgen.1000450.s005.tif]
